# The influence of birth order and number of siblings on adolescent body composition: evidence from a Brazilian birth cohort study

**DOI:** 10.1017/S0007114515001488

**Published:** 2015-06-15

**Authors:** Fernanda de Oliveira Meller, M. C. F. Assunção, A. A. Schäfer, C. L. de Mola, A. J. D. Barros, D. L. Dahly, F. C. Barros

**Affiliations:** 1 Post-Graduate Program in Epidemiology, Federal University of Pelotas, Pelotas, Brazil; 2 Department of Epidemiology and Public Health, University College Cork, Cork, Republic of Ireland

**Keywords:** Birth order, Siblings, Body composition, Adolescents, Cohort studies

## Abstract

The aim of this study was to estimate the association between birth order and number of siblings with body composition in adolescents. Data are from a birth cohort study conducted in Pelotas, Brazil. At the age of 18 years, 4563 adolescents were located, of whom 4106 were interviewed (follow-up rate 81·3 %). Of these, 3974 had complete data and were thus included in our analysis. The variables used in the analysis were measured during the perinatal period, or at 11, 15 and/or 18 years of age. Body composition at 18 years was collected by air displacement plethysmography (BOD POD^®^). Crude and adjusted analyses of the association between birth order and number of siblings with body composition were performed using linear regression. All analyses were stratified by the adolescent sex. The means of BMI, fat mass index and fat-free mass index among adolescents were 23·4 (sd 4·5) kg/m^2^, 6·1 (sd 3·9) kg/m^2^ and 17·3 (sd 2·5) kg/m^2^, respectively. In adjusted models, the total siblings remained inversely associated with fat mass index (β = − 0·37 *z*-scores, 95 % CI − 0·52, − 0·23) and BMI in boys (β = − 0·39 *z*-scores, 95 % CI − 0·55, − 0·22). Fat-free mass index was related to the total siblings in girls (β = 0·06 *z*-scores, 95 % CI − 0·04, 0·17). This research has found that number of total siblings, and not birth order, is related to the fat mass index, fat-free mass index and BMI in adolescents. It suggests the need for early prevention of obesity or fat mass accumulation in only children.

Like most places in the world, the total fertility rate in Brazil is low, and continuing to decline. The average number of births per woman was 2·3 in the year 2000 and 1·9 in 2010^(^ ^1^ ^,^ ^2^ ^)^. Recent studies suggest that the consequent changes in family structure may influence obesity risk in adolescence^(^ ^3^ ^,^ ^4^ ^)^. Some have observed that only children have higher BMI, greater fat mass, and are more likely to be overweight or obese^(^ ^5^ ^–^ ^7^ ^)^. The influence of birth order on the occurrence of obesity has also been investigated, but the findings are inconsistent in previous studies. While some authors showed higher BMI and greater fat mass between firstborn adolescents^(^ ^8^ ^–^ ^11^ ^)^, others have not observed such associations^(^ ^6^ ^,^ ^12^ ^,^ ^13^ ^)^. Studies have also shown that a larger number of siblings is associated with lower prevalence of obesity in adolescence^(^ ^3^ ^,^ ^4^ ^)^, regardless of whether siblings are younger or older^(^ ^4^ ^)^.

There are both social and biological explanations of how birth order and/or number of siblings might influence adolescent body composition. Lower-birth-order infants tend to be smaller at birth than later-born infants^(^ ^14^ ^–^ ^16^ ^)^ and more likely to experience catch-up growth, a pattern of growth associated with obesity risk^(^ ^13^ ^)^. However, lower-birth-order children also tend to have fewer siblings, which might reduce their opportunities for playing games and other physical activities^(^ ^17^ ^)^. It is thus critically important to better understand the relative importance of both birth order and number of siblings, a challenge few previous studies have addressed.

To help fill this gap, we used data from the 1993 Birth Cohort of Pelotas, Brazil, which included detailed information on family structure, to evaluate the association between both birth order and number of siblings with body composition in 18-year-old adolescents.

## Methods

### Study design and sample

Data are from a population-based birth cohort study located in Pelotas, Brazil. Between 1 January and 30 December, 1993, all maternity hospitals in the city were visited daily and 5265 births from woman living in the city were recorded. Of this, 5249 agreed to take part in the longitudinal study. Mothers and their infants have since been followed up on numerous occasions. Visits to the full cohort took place at 11, 15 and 18 years of age. Topic-specific sub-studies were conducted at the ages of 4, 6, 9, 11, 13 and 18 years. More detailed information about the study can be found in specific methodological publications^(^ ^18^ ^,^ ^19^ ^)^.

To be included in the sample used in this analysis, individuals must have participated in the follow-ups at ages 11, 15 and 18 years, and have information on the perinatal exposures (birth order), at age 15 years (number of younger siblings and total siblings) and on the outcomes at age 18 years (BMI, fat mass and fat-free mass). Individuals who at 18 years of age were pregnant or suspected of being pregnant, in a cast, or using a wheelchair were excluded from the sample.

### Measurements

Body composition at 18 years of age was assessed by fat mass index (fat mass in kg divided by height in m^2^) and fat-free mass index (fat-free mass in kg divided by height in m^2^), both collected by air displacement plethysmography (BOD POD^®^), using the Siri equation. These indices include the height of the individual in the calculations, and thus improving interpretation in adolescents with different heights^(^ ^20^ ^)^. Moreover, the BMI (weight in kg divided by height in m^2^) was also assessed at the age of 18 years.

For the examination, the young individuals were attired in a top and shorts made of average compression spandex, and a silicone cap with good grip on the head.

Height was measured using an aluminium stadiometer with size of 2 m and precision of 1 mm; and weight was measured with an electronic scale connected to the BOD POD^®^. All measurements were taken by trained interviewers.

Data collection on major exposures was performed by asking the mother two questions. For data on birth order, we asked ‘How many times have you ever been pregnant including this pregnancy?’, and to collect information on number of younger siblings, we asked ‘Have you had any children after (name)? How many?’ Then, the two responses were combined to build the total siblings variable.

The first question was made during the perinatal follow-up, and the second at the 15-year-old follow-up.

### Analytical methods

The main exposures used in the analyses were birth order (dummy-variable coded as first born, 0; second born, 1; third born, 2; and fourth born or more, 3), number of younger siblings and total siblings (each dummy-variable coded for 0, 1, 2, and more than 3 siblings). The outcomes BMI, fat mass index and fat-free mass index were assessed as continuous variables in kg/m^2^ and standardised as *z*-scores.

For controlling possible confounding factors, the following variables were included in the analysis: family income (in minimum wages), maternal education (in completed years), presence of the father (yes/no), maternal skin colour (white/black/other), maternal age at birth (in years), maternal height (in cm), gestational weight gain (in g), pregnancy smoking consumption (yes/no), pregnancy alcohol consumption (yes/no), gestational systemic hypertension (yes/no) and gestational diabetes (yes/no).

At the age of 18 years, 4563 adolescents were located, of whom 4106 were interviewed and 3974 adolescents were included in our analysis with complete data on body composition. Those who completed the interviews, added to those known to have died, represented 81·3 % of the original cohort. Of those located, 127 (2·3 %) refused to participate in the study and 330 (7·2 %) were considered losses, 196 were found living in other cities and were not interviewed. The maternal height variable had the maximum percentage of unknown observations (7·9 %).

Descriptive analysis of the exposures and outcomes were performed, presenting the absolute and relative frequencies of categorical variables, and measures of central tendency and dispersion for continuous variables.

Later, linear regression models were used to estimate associations between exposures (birth order, number of younger siblings and total siblings) and outcomes (BMI, fat mass index and fat-free mass index). We report crude estimates, as well as those adjusted for the potential confounders described earlier. All analyses were sex stratified.

**Fig. 1:**
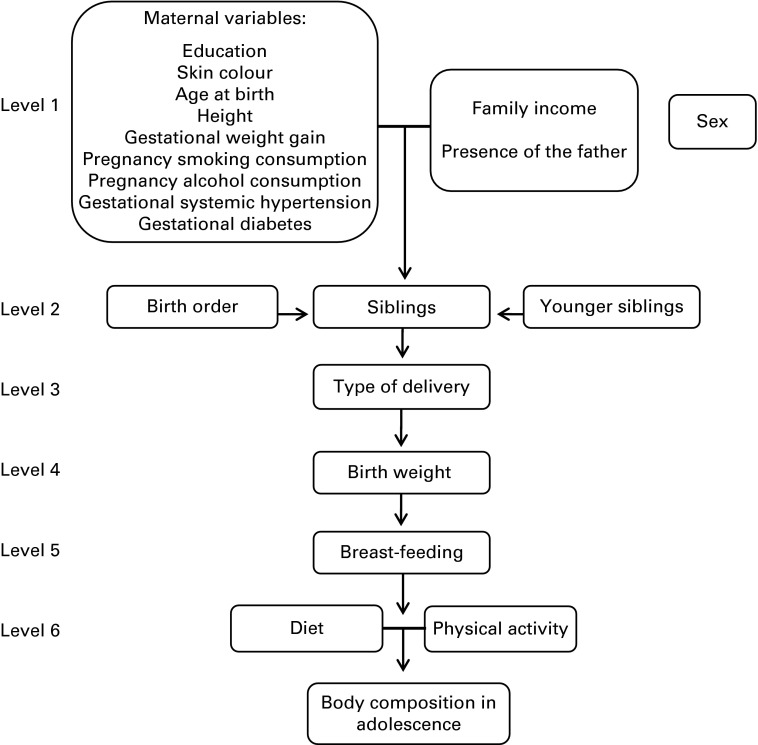
Conceptual model of association between birth order, number of siblings and body composition at 18 years of age. The 1993 Pelotas Birth Cohort, Brazil.

To evaluate the relative importance of birth order and number of siblings, we compared the fit of three different models based on their respective *R* ^2^, adjusted *R* ^2^, Akaike's information criterion and mean squared error. In model 1, only birth order and number of younger siblings were included as independent variables. In model 2, we included these variables plus potential confounders (Fig. 1). Model 3 included the total siblings (which is equal to the birth order plus the number of younger siblings, less one), and the confounding variables of model 2. The purpose of this third model was to evaluate if the position of the adolescent in the family influences the body composition, or whether this association is rather due to the number of siblings. The model that presented the best fit was the one with higher values of *R* ^2^ and adjusted *R* ^2^, and lower values of Akaike's information criterion and mean squared error.

### Ethical considerations

The present study is part of the 18-year-old follow-up in the 1993 birth cohort, titled ‘Early and contemporary influences on body composition, human capital, mental health and complex chronic diseases precursors in the 1993 birth cohort of Pelotas, Brazil’, which was approved by the Ethics Committee of the Medicine School of the Federal University of Pelotas in the official letter numbered 05/11.

All participants signed an informed consent form before the procedures, interviews and examinations in both follow-ups. At the age of 18 years, body composition examinations did not present health risks to participants; nevertheless, girls were always asked about the possibility of pregnancy. Pregnant women and those suspected of being pregnant did not perform the tests.

## Results

Characteristics of sample studied are shown in Table 1. Most of the adolescents were the firstborns (39·8 %), and at the age of 15, 30·8 % had one sibling. Regarding the presence of the father, 11·9 % of adolescents had no father living in the house when they were born. In relation to the mothers, one-third reported smoking during pregnancy (32·9 %) and a small proportion reported drinking alcohol during pregnancy (5·2 %). The average maternal education at birth was about 7 (sd 3·5) years and maternal age at birth was 26 (sd 6·4) years on average. The median family income was 2·6 times (interquartile range 1·5–4·6) the minimum wage.

**Table 1:** Characteristics of sample according to the variables studied (The 1993 Pelotas Birth Cohort, Brazil) (Number of participants and percentages; mean values and standard deviations; median values and interquartile ranges (IQR))

	Total	Male	Female
Variables	*n*	%	*n*	%	*n*	%
Birth order						
First born	1634	39·8	791	39·3	843	40·4
Second	1225	29·9	603	29·9	622	29·8
Third	652	15·9	311	15·4	341	16·3
Fourth or later	592	14·4	311	15·4	281	13·5
Younger siblings						
0	1621	41·5	770	41·2	851	42·0
1	1300	33·4	617	33·0	683	33·7
2	545	14·0	286	15·3	259	12·8
≥ 3	431	11·1	197	10·5	234	11·5
Total siblings						
0	470	12·1	216	11·6	254	12·5
1	1198	30·8	560	30·0	638	31·6
2	963	24·7	466	25·0	497	24·5
≥ 3	1263	32·4	626	33·4	637	31·4
Maternal skin colour						
White	3155	76·9	1562	77·4	1593	76·3
Black	763	18·6	361	17·9	402	19·3
Other	186	4·5	95	4·7	91	4·4
Pregnancy smoking consumption						
Yes	1349	32·9	644	31·9	705	33·8
No	2757	67·1	1374	68·1	1383	66·2
Pregnancy alcohol consumption						
Yes	212	5·2	91	4·5	121	5·8
No	3894	94·8	1927	95·5	1967	94·2
Gestational systemic hypertension						
Yes	631	15·7	332	16·8	299	14·6
No	3398	84·3	1645	83·2	1753	85·4
Gestational diabetes						
Yes	108	2·7	51	2·6	57	2·8
No	3911	97·3	1918	97·4	1993	97·2
Presence of the father						
Yes	3618	88·1	1766	87·5	1852	88·7
No	488	11·9	252	12·5	236	11·3
Maternal education (completed years)	4099		2014		2085	
Mean	6·8		6·8		6·7	
sd	3·5		3·5		3·5	
Maternal age at birth (years)	4105		2017		2088	
Mean	26·1		26·1		26·1	
sd	6·4		6·5		6·3	
Maternal height* (cm)	3780		1832		1948	
Mean	157·7		157·6		157·8	
sd	6·7		6·3		7·0	
Gestational weight gain (g)	3953		1940		2013	
Mean	11·7		11·9		11·6	
sd	5·9		5·9		5·8	
Family income (MMW)	4035		1989		2046	
Median	2·6		2·5		2·8	
IQR	1·5–4·6		1·5–4·5		1·5–4·8	

MMW, monthly minimum wages.

* Maximum percentage of unknown observations: (*n* 326; 7·9 %) for the maternal height variable.

Table 2 shows the mean and standard deviations of BMI, fat mass index and fat-free mass index among male and female adolescents, and in the total population. These averages were 23·4 (sd 4·5) kg/m^2^, 6·1 (sd 3·9) kg/m^2^ and 17·3 (sd 2·5) kg/m^2^, respectively. Although BMI was the same for both sexes, girls had an average fat mass index almost two times higher than boys, while the mean fat-free mass index was higher in males. The prevalence of overweight and obesity in the adolescents, using the BMI-for-age WHO criterion, was 17·2 (95 % CI 16·0, 18·3) % and 10·1 (95 % CI 9·2, 11·0) %, respectively.

**Table 2:** Description of the sample according to outcomes studied (The 1993 Pelotas Birth Cohort, Brazil) (Mean values and standard deviations)

	Total (*n* 3974)	Male (*n* 1973)	Female (*n* 2001)
Variables	Mean	sd	Mean	sd	Mean	sd
BMI (kg/m^2^)	23·4	4·5	23·4	4·2	23·5	4·8
Fat mass index (kg/m^2^)	6·1	3·9	4·2	3·1	8·0	3·6
Fat-free mass index (kg/m^2^)	17·3	2·5	19·1	1·9	15·5	1·6

Table 3 presents the fit of the three models analysed, for each of the three outcomes investigated. In general, model 3 showed the best fit for each outcome, and was more parsimonious than model 2. While models 1 and 2 indicated that both birth order and number of younger siblings were associated with the outcome (data not shown), the better fit of model 3 led us to conclude that the total siblings was the key influence on fat mass index, fat-free mass index and BMI, rather than the position that the adolescents occupy among siblings.

**Table 3:** Evaluation of the goodness of fit of analysed models (The 1993 Pelotas Birth Cohort, Brazil)

	*n*	*R* ^2^	Adjusted *R* ^2^	AIC	MSE
Outcome: fat mass index					
Model 1*	3788	0·01	0·01	10 720·28	1·00
Model 2†	3345	0·02	0·01	9471·34	0·99
Model 3‡	3345	0·02	0·01	9465·31	0·99
Outcome: fat-free mass index					
Model 1*	3788	0·003	0·002	10 777·17	1·00
Model 2†	3345	0·02	0·01	9503·96	1·00
Model 3‡	3345	0·02	0·01	9506·15	1·00
Outcome: BMI					
Model 1*	3788	0·005	0·004	10 770·88	1·00
Model 2†	3345	0·02	0·01	9525·77	1·00
Model 3‡	3345	0·02	0·01	9523·82	1·00

AIC, Akaike's information criterion; MSE, mean squared error.

* Birth order and total siblings adjusted one for each other.

† Birth order and younger siblings adjusted for confounding variables: family income, maternal education, presence of the father, maternal skin colour, maternal age at birth, maternal height, gestational weight gain, pregnancy smoking consumption, pregnancy alcohol consumption, gestational systemic hypertension and gestational diabetes.

‡ Total siblings adjusted for confounding variables: family income, maternal education, presence of the father, maternal skin colour, maternal age at birth, maternal height, gestational weight gain, pregnancy smoking consumption, pregnancy alcohol consumption, gestational systemic hypertension and gestational diabetes.

**Fig. 2:**
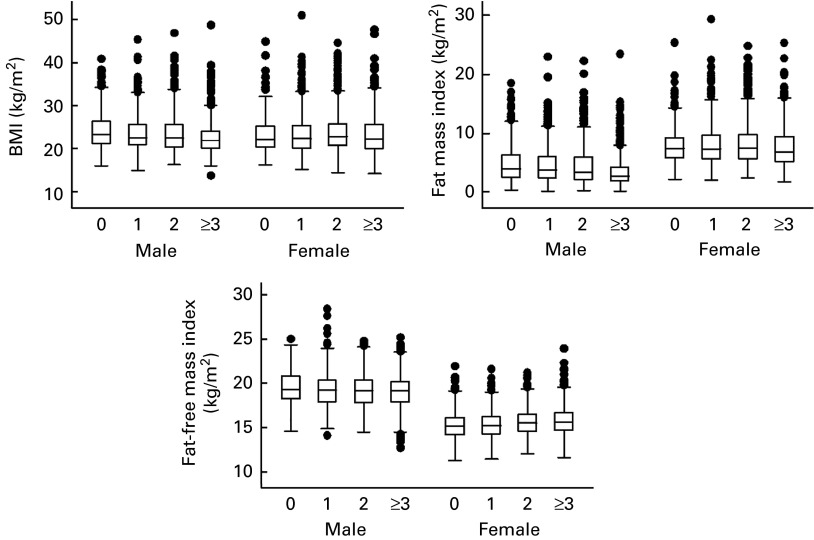
Distributions of BMI, fat mass index and fat-free mass index, across total siblings, by sex. The 1993 Pelotas Birth Cohort, Brazil.

The distributions of BMI, fat mass index and fat-free mass index, across total siblings, by sex are presented in Fig. 2. Boys who had three or more siblings had lower BMI mean (22·6, 95 % CI 22·3, 22·9) compared to those of only children (24·4, 95 % CI 23·8, 25·1). Fat mass index was associated to total siblings in both sexes. Adolescents with three or more siblings had lower fat mass index mean than those who had no siblings. Girls who had three or more siblings had higher fat-free mass index mean (15·7, 95 % CI 15·5, 15·8) than those who had none (15·3, 95 % CI 15·1, 15·5).

Table 4 shows the crude and adjusted analyses of the association between total siblings and the outcomes studied according to sex. After adjustment for possible confounders (family income, maternal education, presence of the father, maternal skin colour, maternal age at birth, maternal height, gestational weight gain, pregnancy smoking consumption, pregnancy alcohol consumption, gestational systemic hypertension and gestational diabetes), the total siblings remained associated with fat mass index in both sexes with an inverse linear trend in males (*P*< 0·001). Boys who had three or more siblings showed a decrease of 0·37 *z*-scores (95 % CI − 0·52, − 0·23) in fat mass index compared to those who had no siblings. Fat-free mass index was associated with the total siblings only in girls (*P*= 0·035). Those with three or more siblings showed a 0·06 increase in *z*-score (95 % CI − 0·04, 0·17) compared to girls who had none. Moreover, the total siblings remained inversely related to BMI in males (*P*< 0·001). Those boys with three or more siblings had a reduction of 0·39 *z*-scores in BMI when compared to an only child (95 % CI − 0·55, − 0·22).

**Table 4:** Crude and adjusted analyses of association between siblings and outcomes (in *z*-score) stratified by sex (The 1993 Pelotas Birth Cohort, Brazil) (Number of participants; β coefficients and 95 % confidence intervals)

	Crude analyses	Adjusted analyses*
	Male	Female	Male	Female
	*n*	β	95 % CI	*P*	*n*	β	95 % CI	*P*	β	95 % CI	Valor *P*	β	95 % CI	*P*
Outcome: fat mass index														
Total siblings			< 0·001†				0·023			< 0·001†			0·010
0	213	Reference		245	Reference		Reference		Reference	
1	553	− 0·13	− 0·26, − 0·01		621	0·04	− 0·09, 0·18		− 0·13	− 0·27, 0·004		0·02	− 0·13, 0·16	
2	460	− 0·14	− 0·27, − 0·01		479	0·08	− 0·06, 0·22		− 0·14	− 0·29, − 0·01		0·05	− 0·10, 0·20	
≥ 3	616	− 0·39	− 0·51, − 0·26		601	− 0·08	− 0·22, 0·06		− 0·37	− 0·52, − 0·23		− 0·14	− 0·30, 0·01	
Outcome: fat-free mass index														
Total siblings			0·164				< 0·001†			0·083†			0·035
0	213	Reference		245	Reference		Reference		Reference	
1	553	− 0·07	− 0·19, 0·05		621	0·01	− 0·09, 0·10		− 0·08	− 0·20, 0·04		− 0·01	− 0·11, 0·09	
2	460	− 0·09	− 0·21, 0·03		479	0·14	0·04, 0·24		− 0·11	− 0·24, 0·01		0·10	− 0·002, 0·21	
≥ 3	616	− 0·13	− 0·24, − 0·01		601	0·15	0·06, 0·25		− 0·12	− 0·25, 0·01		0·06	− 0·04, 0·17	
Outcome: BMI														
Total siblings			< 0·001†				0·145			< 0·001†			0·067
0	213	Reference		245	Reference		Reference		Reference	
1	553	− 0·15	− 0·30, − 0·01		621	0·04	− 0·12, 0·20		− 0·16	− 0·31, 0·001		0·01	− 0·15, 0·18	
2	460	− 0·17	− 0·32, − 0·02		479	0·15	− 0·01, 0·31		− 0·19	− 0·36, − 0·03		0·10	− 0·07, 0·27	
≥ 3	616	− 0·41	− 0·55, − 0·26		601	0·02	− 0·14, 0·18		− 0·39	− 0·55, − 0·22		− 0·09	− 0·26, 0·09	

* For family income, maternal education, presence of the father, maternal skin colour, maternal age at birth, maternal height, gestational weight gain, pregnancy smoking consumption, pregnancy alcohol consumption, gestational systemic hypertension and gestational diabetes.

† Linear trend test.

## Discussion

An important result of the present study was the association between number of total siblings and fat mass index in adolescents of both sexes. The smaller the number of total siblings, the greater the fat mass index, even after adjustment for possible confounding factors. Furthermore, it was shown that the number of total siblings remained directly associated with the fat-free mass index in girls, even after adjustment for potential confounders. However, it is important to consider both associations in the girls were weak, although significant.

Similar results were found in a study in India with women only, which showed a negative correlation between number of siblings and both fat mass and fat-free mass. However, the Indian study was not adjusted for potential confounding factors and body composition was assessed by skinfold thickness^(^ ^21^ ^)^. Studies that assess the body composition of adolescents using reference measurement methods are still rare in the literature, which hinders the comparison of results.

Concerning BMI, it was shown that this variable remained inversely associated with the number of total siblings in males in the adjusted analysis. One possible explanation for this association is that the greater number of siblings leads to an increase in family size, which has an inverse effect on family income and, consequently, on obesity. According to a study conducted in Brazil, there is a direct association between income and obesity in adolescents of both sexes^(^ ^22^ ^)^.

Corroborating the results of the present study, a longitudinal study conducted in the USA showed that only children had higher mean BMI compared to those who had at least two brothers, even after adjustment for possible confounding factors^(^ ^5^ ^)^. Other studies analysing overweight and/or obesity in adolescents, using different methods^(^ ^23^ ^–^ ^25^ ^)^, observed inverse associations between number of siblings and overweight and/or obesity, with only children having a higher prevalence of overweight and obesity when compared with individuals who had siblings^(^ ^3^ ^,^ ^4^ ^,^ ^6^ ^,^ ^7^ ^,^ ^26^ ^)^. In contrast, Hesketh and colleagues^(^ ^27^ ^)^ showed no association between number of siblings and overweight in Chinese adolescents. Results of stratified analysis by sex are presented for one study only^(^ ^4^ ^)^, which hinders the comparison of findings.

Some limitations in the present study should be highlighted. First, as with any longitudinal study, potential selection biases due to loss to follow-up are a substantial limitation. To help address this concern, we compared the sample participants with the original participants examined in 1993. Adolescents with worse socioeconomic and nutritional profiles were slightly less likely to be followed up. Socioeconomically intermediate participants were more likely to located as compared with very poor or very rich adolescents (81·8 *v.* 75·6 and 76·1 %, respectively). Furthermore, participants whose mothers had no schooling were less likely to be followed up compared to those whose mothers had 9 or more years of schooling (69·4 *v.* 77·5 %). However, the magnitude of such differences is modest, therefore minimising the likelihood of bias^(^ ^18^ ^)^.

Another limitation is the lack of information on the number of siblings of adolescents at 18 years of age, which limits us to the information collected at the 15-year-old follow-up. Finally, the measurements of body composition are only available at age 18 years; therefore, we were not able to investigate the development of body composition across the entire life course.

Strengths of the present study include its prospective design, the use of air displacement plethysmography (BOD POD^®^) as a method for assessing body composition, and the high follow-up rate at age 18 years (81·3 %) ensuring the representativeness of the sample despite some small differences between study participants and those lost to follow-up.

In conclusion, only children of both sexes are more likely to have higher fat mass index compared to those who have siblings. Girls who have no siblings present the lowest levels of fat-free mass, while only boy children have greater BMI.

Given the reduction in the fertility rate in Brazil and in the world^(^ ^1^ ^,^ ^2^ ^,^ ^28^ ^,^ ^29^ ^)^ and the larger number of couples choosing to have an only child^(^ ^29^ ^)^, our research further highlights the need for prevention of obesity or fat mass accumulation in adolescents who are only children.
